# Outcome After Treatment With 15% Trichloroacetic Acid Peeling After Facial Black Powder Injury

**DOI:** 10.1093/asjof/ojac056

**Published:** 2022-06-24

**Authors:** Franzisca Ulrich, Leslie Elahi, Severin Alexander Rossi, Joachim Meuli, Wassim Raffoul

**Affiliations:** Department of Plastic, Reconstructive and Hand Surgery, University Hospital of Lausanne (CHUV), Lausanne, Switzerland; Department of Plastic, Reconstructive and Hand Surgery, University Hospital of Lausanne (CHUV), Lausanne, Switzerland; Department of Plastic, Reconstructive and Hand Surgery, University Hospital of Lausanne (CHUV), Lausanne, Switzerland; Department of Plastic, Reconstructive and Hand Surgery, University Hospital of Lausanne (CHUV), Lausanne, Switzerland; Department of Plastic, Reconstructive and Hand Surgery, University Hospital of Lausanne (CHUV), Lausanne, Switzerland

## Abstract

Explosion-related facial black powder injuries are difficult to treat due to traumatic tattooing of powder residue. A common treatment protocol consists of immediate debridement using a variety of surgical techniques with no other adjuncts. The results are often mediocre because of scarring or powder remnants on the skin. The authors present a patient who suffered innumerable black powder tattoos to the face after a cannon explosion treated by initial debridement and serial treatments of 15% trichloroacetic acid peelings with a highly satisfactory result.

**Level of Evidence: 5:**

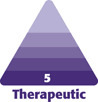

Traumatic tattooing is a challenging entity, especially in exposed skin areas such as the face or hands. Literature is scarce, and various articles mention different treatments, ranging from sole initial surgical debridement, excision by 11-mm blade to serial dermabrasions.^1-4^ Unfortunately, results are oftentimes unsatisfactory due to ice pick scarring or remaining residue of the traumatic tattooing. Patients also often require lengthy social eviction after deep dermabrasion due to erythema or swelling, additionally requiring adjunct antibiotic treatments and long healing.^5^

In our tertiary burn center, we adopted a standard treatment for burn patients with soot residue in the face: initial scrubbing under general anesthesia, with additional curettage where necessary. Additionally, our esthetic clinic treats patients seeking skin improvement by resurfacing with 15% trichloroacetic acid (TCA) peelings, which show highly satisfactory results with minimal downtime. TCA is a chemical peeling used for skin rejuvenation by coagulation of proteins in the epidermis, papillary, and reticular dermis, leading to a cutaneous regeneration.

We present a patient who suffered facial black powder injury after a cannon blast, with traumatic tattooing of soot to the face. We treated him with initial surgical debridement and additional serial treatments with 15% TCA peeling. The results were highly satisfactory. To our knowledge, this treatment plan in case of facial traumatic tattooing has not previously been reported.

## CASE PRESENTATION

**Figure 1.**
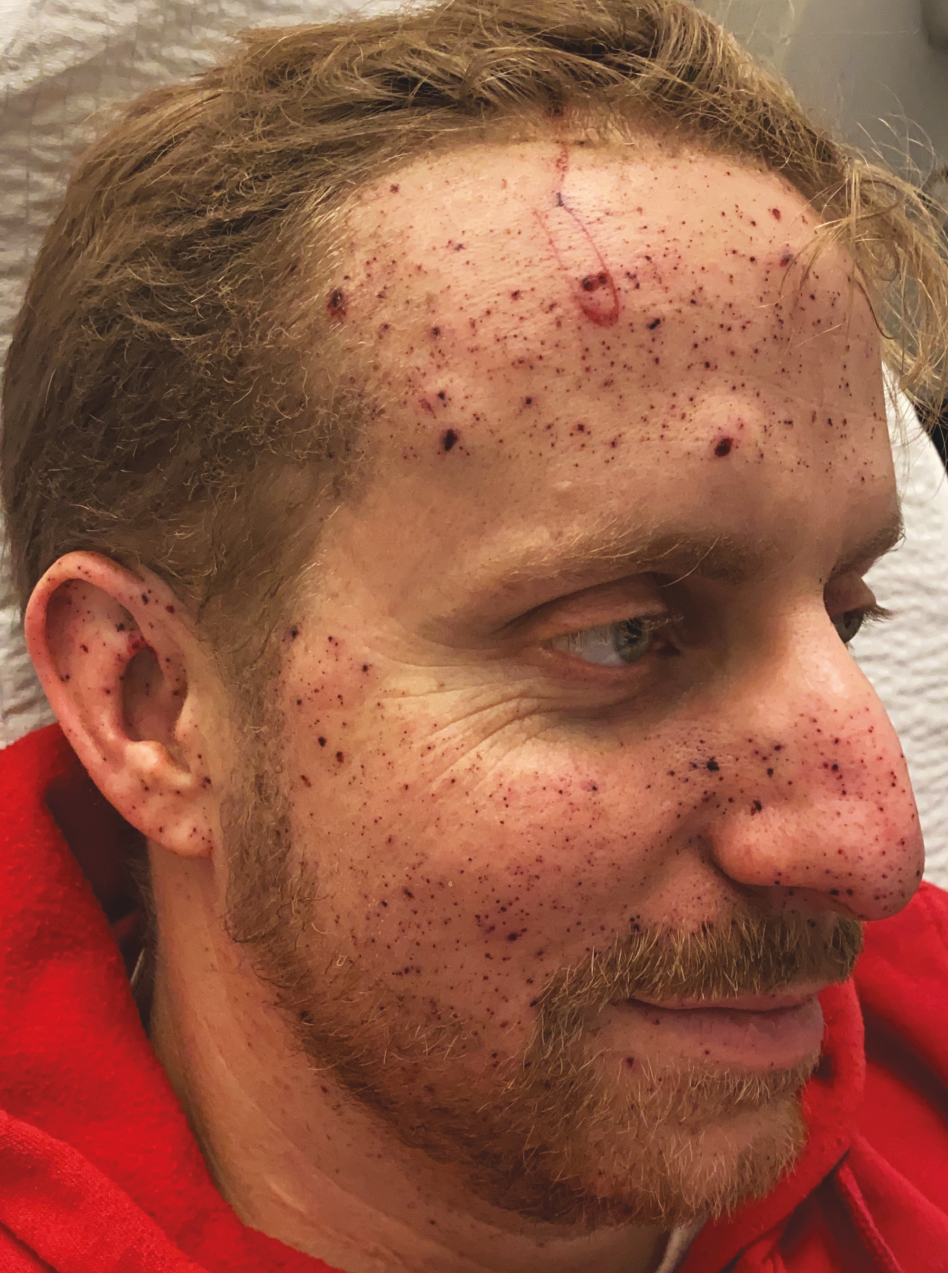
A 34-year-old male patient shown on the first day after trauma.

A 34-year-old male presented with a superficial burn and traumatic black powder tattooing after a cannon explosion (Figure 1). The patient had no comorbidities and never had any other facial treatment or surgery. On examination, the patient showed a multitude of millimetric traumatic tattooing covering the whole left half of the face. The patient wore sunglasses during the explosion; his eyes were, therefore, not injured. Also, the patient wore a beard, protecting most of his lower face.

The initial treatment of the injury comprised urgent debridement in the operating room within the first 24 hours after the explosion. We used a 3-mm sinus lift curette and a 20-gauge needle to debride the black powder remnants deep in the dermis and hypodermis. Wounds larger than 1 mm with gun powder residue were been reopened and cleared. The typical gray film and superficial black powder lesions in the epidermis have been left untouched in order to avoid the typical “ice pick” scarring occurring with heavy-handed debridement. We instructed the patient after surgery to keep his skin moist by application of dexpanthenol cream (Bepanthen, Bayer AG, Leverkusen, Germany).

**Figure 2.**
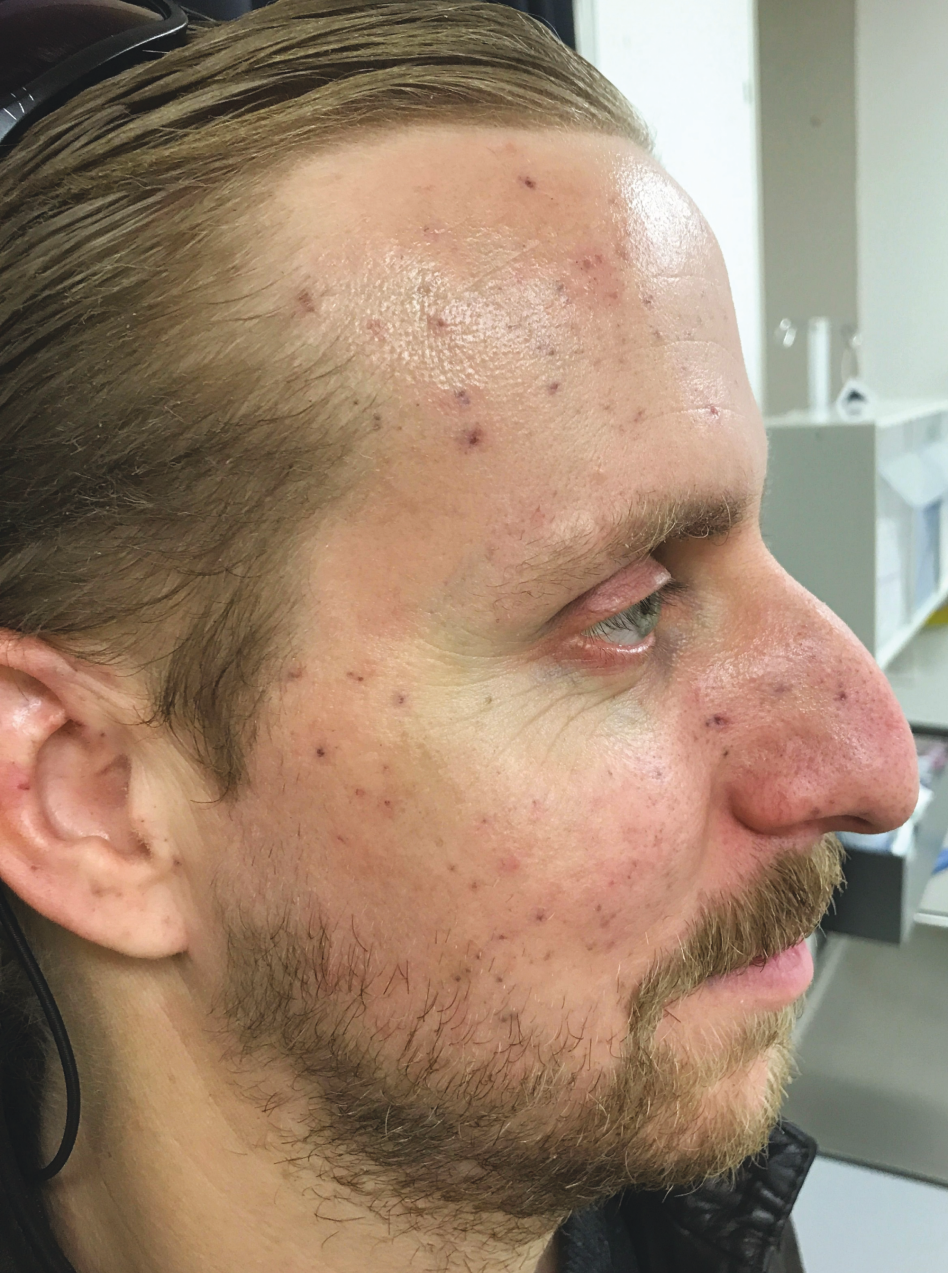
The same 34-year-old male patient from Figure 1 shown 7 days post-surgery.

**Figure 3.**
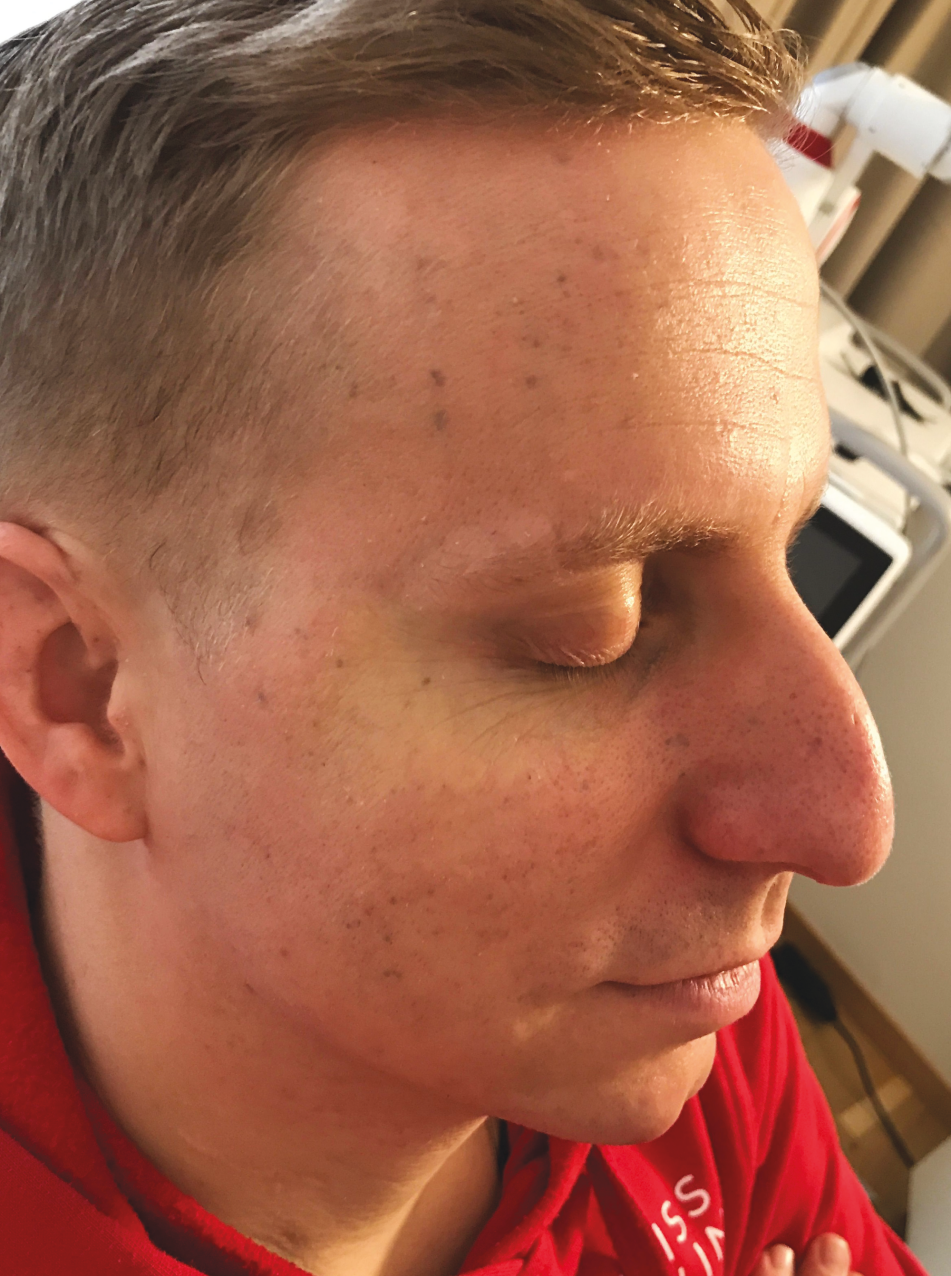
The 34-year-old male patient from Figures 1, 2 shown 3 months post-surgery.

One week after surgery, healing was progressing well and only isolated crusts of the deeper wounds remained (Figure 2). In order to give the skin time to heal, as well as to attain a “steady state” and evaluate powder remnants, we delayed further treatments for 3 months (Figure 3).

**Figure 4.**
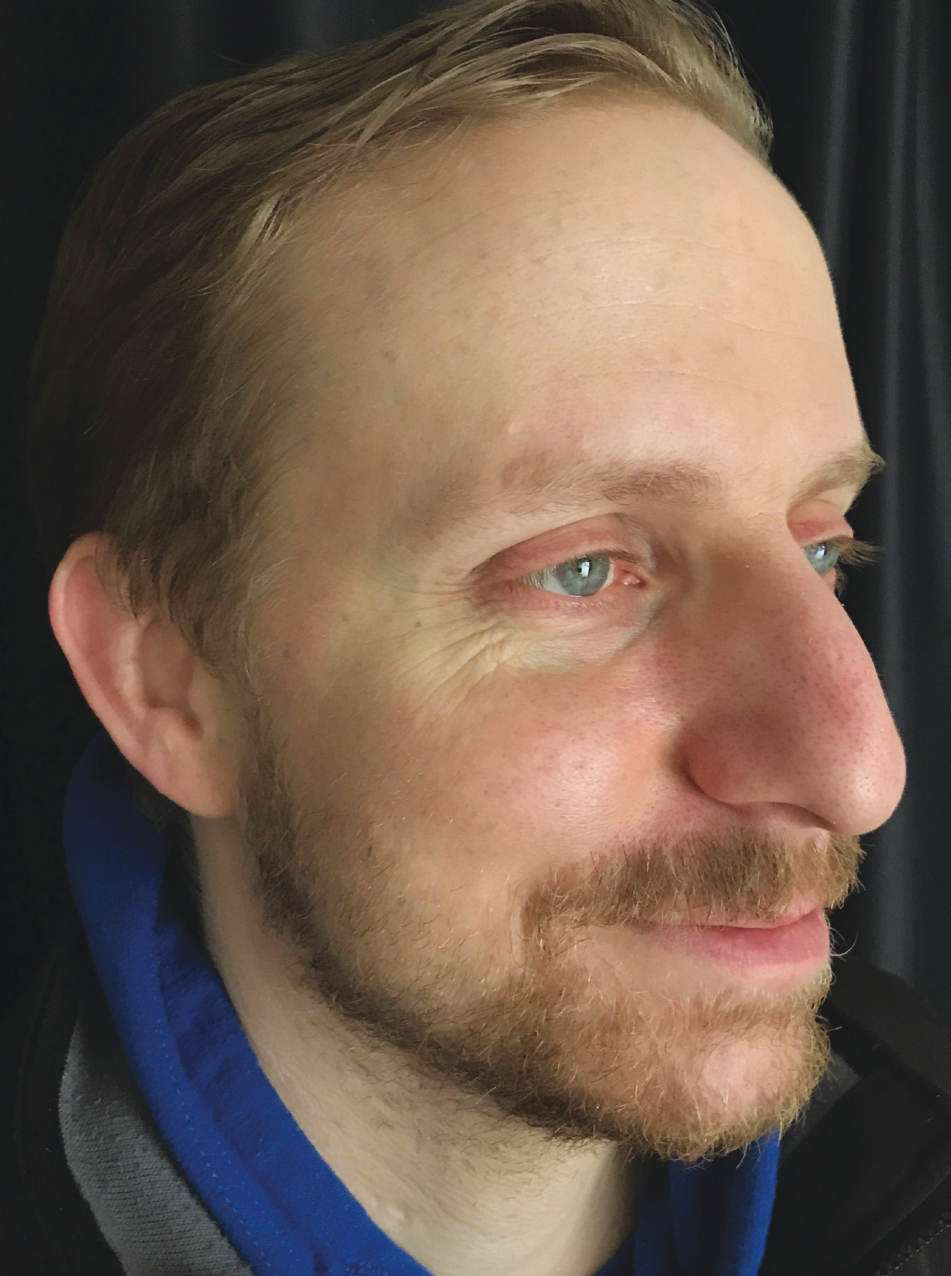
The final results shown on the 34-year-male patient from Figures 1-3 after 4 sessions of trichloroacetic acid peeling.

We then started chemical peeling with 15% TCA (Easy TCA, Skin Tech Pharma Group, Girona, Spain). We prepared the skin with chlorhexidine digluconate (Hibidil CPS Cito Pharma Services GmbH, Uster, Switzerland) before the treatment. We applied 3 layers of 15% TCA until the appearance of “clouds,” indicating a “medium” peeling down to the zone of Grenz. The first layer showed a typical erythema indicating penetration of the chemical in the stratum corneum. The second layer showed white dots, indicating the crossing of the epidermis down to the basal layer. The third layer showed white “clouds,” indicating penetration to our mark in the Grenz zone. We performed a series of 4 sessions with a 7-day interval between the treatments. Preliminary results were visible after the first session, the patient mentioning an improvement in skin tone and decreased amount of black and grayish skin. We saw an improvement in the quantity and hue of the traumatic tattoos and the aspect of the skin in general (Figure 4). Every successive peeling session further improved skin tone and quality. In the end, traumatic pigmentation had vanished nearly completely. 

## DISCUSSION

Traumatic tattooing occurs by accidental deposition of pigmented particles in the dermis. Carbon is the most common material deposited, often after explosions, and gives a black or blue color, depending on the depth of penetration in the dermis. Dermabrasion and direct excision have been described as treatments yielding acceptable results; however, pictures often show either ice pick scaring or tattoo remnants.^1,^^5-7^ Laser treatment has also been described with good clinical outcomes, its downside is that it cannot be used on every skin type, and it is not easily and training costs are high.^8-12^

TCA peeling allows for a less aggressive pre-debridement in the OR with nearly no recovery time needed, as only large (ie, >1 mm) deep lesions in the reticular dermis and hypodermis need to be debrided. Therefore, millimetric instruments can be used to remove the remnants with the advantage of minimal visual scarring. The more superficial lesions can be treated secondarily with the TCA peeling. The advantage of a 15% TCA peeling is its safety and ability to be applied on a large surface. Higher concentrations are known to be used in smaller areas. Other peelings such as alpha hydroxy acid peels or phenol peels either do not reach deeper skin layers or, on the contrary, target deeper layers with significant downtimes and higher complication rates. In the context of explosion injury, we wanted to keep potential complications and time of convalescence to a minimum. The depth of the peeling is influenced by the number of applied layers. A 15% TCA peeling can treat the skin as deep as the papillary layer, shown by a uniform white discoloration of the skin during application. Within the first week after a TCA peel, patients undergo progressive desquamation from day 3 to day 8, revealing a renovated skin. During this period, it is imperative to use moisturizing creams and sun protection. After the desquamation phase, it is not necessary to use a hydrating cream anymore, but sunscreen remains mandatory. The peeling can be repeated after a week.

Patients should be advised for eventual erythema or pruritus, rebound pigmentations, or dyschromia. However, rebound pigmentations and dyschromia are rare,^13^ mostly associated with deeper peels (down to the papillary and reticular dermis), and are, therefore, rare in peels superficial to the zone of Genz. By performing several sessions down to Grenz’s zone and the papillary dermis, gunpowder remnants, or indeed any other tattooing particle, may be removed in a gentle, controlled manner, and by inducing skin rejuvenation, it is also beneficial for the healing process.

## CONCLUSIONs

Gunpowder injuries with subsequent traumatic tattooing are difficult to treat. Therefore, our tertiary multidisciplinary clinic treating burn patients and aesthetic patients alike is developing a better treatment option for these challenging, often stigmatizing injuries. By using only small instruments (0.9 to 3 mm) during initial debridement, visual scarring was prevented. To avoid dermabrasion with a long recovery time and social eviction, 15% TCA peelings can be used to improve the overall skin aspect. TCA is easy to use, has a small complication profile, and is convenient, readily available, and cheap. It is, therefore, a good alternative to laser treatment in this indication.

## References

[1] Sun B, Guan W. Treating traumatic tattoo by micro-incision. Chin Med J (Engl). 2000;113(7):670-671. 11776045

[2] Parsons RW . The management of traumatic tattoos. Clin Plast Surg. 1975;2(4):517-522. doi: 10.1016/s0094-1298(20)30268-6 1157448

[3] Notaro WA . Dermabrasion for the management of traumatic tattoos. J Dermatol Surg Oncol. 1983;9(11):916-918. doi: 10.1111/j.1524-4725.1983.tb01039.x 6630705

[4] Kurokawa M, Isshiki N, Taira T, Matsumoto A. The use of microsurgical planning to treat traumatic tattoos. Plast Reconstr Surg. 1994;94(7):1069-1072. doi: 10.1097/00006534-199412000-00025 7972463

[5] Cronin ED, Haber JL. A new technique of dermabrasion for traumatic tattoos. Ann Plast Surg. 1996;36(4):401-402. doi: 10.1097/00000637-199604000-00014 8728585

[6] Horowitz J, Nichter LS, Stark D. Dermabrasion of traumatic tattoos: simple, inexpensive, effective. Ann Plast Surg. 1988;21(3):257-259. doi: 10.1097/00000637-198809000-00012 3223706

[7] Zook EG . Care of the traumatic tattoo. Med times. 1974;102(12):90-92, 1-5. 4437363

[8] Achauer BM, Nelson JS, Vander Kam VM, Applebaum R. Treatment of traumatic tattoos by Q-switched ruby laser. Plast Reconstr Surg. 1994;93(2):318-323. doi: 10.1097/00006534-199402000-00014 8310023

[9] Haywood RM, Monk BE, Mahaffey PJ. Treatment of traumatic tattoos with the Nd YAG laser: a series of nine cases. Br J Plast Surg. 1999;52(2):97-98. doi: 10.1054/bjps.1998.3054 10434886

[10] Bonan P, Bassi A, Bruscino N, et al Combined pulsed dye laser and Q-switched Nd:YAG laser intraumatic facial tattoo removal: a case series. Dermatol Ther. 2019;32(5):e13069. doi: 10.1111/dth.13069 31430015

[11] Jeon H, Geronemus RG. Successful treatment of a traumatic tattoo in a pediatric patient using a 755-nm picosecond laser. Pediatr Dermatol. 2018;35(6):e430-e431. doi: 10.1111/pde.13668 30303558

[12] Seitz AT, Grunewald S, Wagner JA, Simon JC, Paasch U. Fractional CO_2_ laser is as effective as Q-switched ruby laser for the initial treatment of a traumatic tattoo. J Cosmet Laser Ther. 2014;16(6):303-305. doi: 10.3109/14764172.2014.956669 25148407

[13] S Sitohang IB, Legiawati L, Suseno LS, Safira FD. Trichloroacetic acid peeling for treating photoaging: a systematic review. Dermatol Res Pract. 2021;2021:3085670. doi: 10.1155/2021/3085670. 34504524 PMC8423570

